# Diversity and role of cave-dwelling hematophagous insects in pathogen transmission in the Afrotropical region

**DOI:** 10.1038/emi.2017.6

**Published:** 2017-04-12

**Authors:** Judicaël Obame-Nkoghe, Eric-Maurice Leroy, Christophe Paupy

**Affiliations:** 1 Laboratoire MIVEGEC, UMR 224-5290 CNRS-IRD-UM, IRD Montpellier, 911, Avenue Agropolis, BP 64501, 34394 Montpellier Cedex 5, France; 2 Centre International de Recherches Médicales de Franceville (CIRMF), BP 769 Franceville, Gabon

**Keywords:** Africa, bat, cave, Diptera, emergence, zoonotic pathogen

## Abstract

The progressive anthropization of caves for food resources or economic purposes increases human exposure to pathogens that naturally infect cave-dwelling animals. The presence of wild or domestic animals in the immediate surroundings of caves also may contribute to increasing the risk of emergence of such pathogens. Some zoonotic pathogens are transmitted through direct contact, but many others require arthropod vectors, such as blood-feeding insects. In Africa, hematophagous insects often play a key role in the epidemiology of many pathogens; however, their ecology in cave habitats remains poorly known. During the last decades, several investigations carried out in Afrotropical caves suggested the medical and veterinary importance particularly of insect taxa of the Diptera order. Therefore, the role of some of these insects as vectors of pathogens that infect cave-dwelling vertebrates has been studied. The present review summarizes these findings, brings insights into the diversity of cave-dwelling hematophagous Diptera and their involvement in pathogen transmission, and finally discusses new challenges and future research directions.

## INTRODUCTION

Caves have been used by earlier human ancestors, and then also by modern humans during the Paleolithic age to face climate hazards or for various shamanic cults. Previous investigations have highlighted the importance of caves for human life, culture and survival.^1^ Currently, caves are becoming more and more anthropized, mainly for resource gathering by the nearby human populations or for economic purposes, such as ecotourism or mining. Therefore, the associated health risks, particularly those related to potential zoonotic pathogens that circulate among cave-dwelling vertebrates, need to be addressed. Contacts between animal reservoirs, such as bats living inside caves, and recipient hosts, such as humans and wild or domestic animals entering or living close to caves, may favor pathogen spillover to new hosts. In specific environmental conditions, these pathogens could easily cross species barriers and cause the emergence of new zoonotic diseases.

Among such pathogens, the most threatening for humans and domestic animals are viruses that, due to their diversity, are responsible for several diseases such as hemorrhagic (for example, Filoviridae), respiratory (for example, coronaviruses), or encephalitic (for example, Henipaviruses) syndromes; bacteria causing bartonellosis (*Bartonella* spp.); and fungi, such as those causing histoplasmosis (*Histoplasma* spp.).^2, 3, 4^ It is currently unknown whether blood parasites housed by cave-dwelling animal reservoirs can develop in humans, but they remain a potential zoonotic risk, as known for viruses. The recent discovery in humans of the monkey parasite *Plasmodium knowlesi* stands out as an example of the potential transfers of animal parasites to humans.^5^

While some zoonotic pathogens can be transferred through direct contact with the animal reservoir, others require blood-feeding arthropods. In caves and in the surrounding areas, humans and animals are almost inevitably exposed to hematophagous arthropods, especially insects. Although hematophagous insects play a key role in the epidemiology of zoonotic pathogens, very little is known about their ecology and distribution in cave ecosystems. Nevertheless, previous investigations have highlighted the great diversity of some insect groups, especially mosquitoes, sand flies, biting midges, bugs and other ectoparasites, in caves worldwide.^6^

In the Afrotropical region, most of the knowledge on cave-dwelling arthropod communities stems from studies carried out in West, Central and East Africa. Although very basic and mainly restricted to insects, these studies are still the reference for hematophagous dipterans, such as mosquitoes (Culicidae), biting midges (Ceratopogonidae), sand flies (Phlebotominae) and bat flies (Nycteribiidae and Streblidae). Members of the Diptera order have been particularly studied because of their importance in human and animal health, but other insect groups, such as bugs and fleas, have also been occasionally detected in African caves.^7^ The present review summarizes the knowledge gained during forty years of investigations on the diversity of hematophagous dipterans living in Afrotropical caves and their involvement in pathogen transmission. It also discusses new challenges and future research directions in this field.

Relevant publications were identified by searching the NCBI PubMed and ISI Web of Knowledge databases and the database of the Office of the Assistant Secretary of Defence for Energy, Installations, and Environment (Armed Forces Pest Management Board (http://www.afpmb.org/content/literature-retrieval-system) using key word combinations that included ‘Diptera', ‘Hematophagous', ‘Cave-dwelling', ‘Afrotropical', ‘Ethiopian Region', ‘Culicidae', ‘Phlebotominae', ‘Ceratopogonidae', ‘Nycteribiidae', ‘Streblidae', ‘Caves', ‘Cave-dwelling bats', ‘Cave-dwelling vectors‘ and ‘Haemosporidia'. Articles in French language were also included because they are particularly numerous on this topic in this part of the world. French articles were retrieved using the Horizon database, hosted by the Institut de Recherche pour le Développement (www.horizon.ird.fr) and the same key word combinations translated in French. Most of the cited literature is available online, but some of the articles can be made available upon request.

### Caves are suitable breeding places for insects

#### The cave physical and micro-climatic features

As in other parts of the world, Afrotropical caves are characterized by stable levels of temperature/relative humidity and the absence of light.^8^ Temperature usually ranges between 22 and 28 °C and relative humidity between 95% and 100%^9^ however, both are modulated by air flows that generate micro-climatic variations according to the cave architecture and especially the size and shape of the internal chambers.^10^ Differences in the rock physical nature and in the seepage or stream water features result in a great variety of caves that are mainly classified in caves formed in non-soluble or in soluble rock.^11^ Caves formed in non-soluble rock are generally of volcanic origin, whereas soluble rock caves are usually found in karst landscapes (that is, areas rich in limestone and characterized by sinks, ravines and underground streams)^11^ or more rarely in areas with lateritic rocks (that is, rocks rich in iron and aluminum), as observed in Gabon.^12^

#### Caves provide ‘board and lodging'

Availability and characteristics of breeding places. In caves, preimaginal development takes place in different breeding sites, from water pools to damp substrata, according to the dipteran group. Mosquitoes breed in residual water contained in rock pools or in shallow pools along the edge of underground rivers.^13^ Larvae of biting midges develop in muddy substrata with a semi-liquid to slightly damp powdery texture.^14^ Sand flies need generally dryer breeding sites and, depending on the species, immature stages can develop deep in the soil or at the surface.^15^ Although the different breading sites have specific features, all contain organic matter to support preimaginal development. As the absence of light excludes the development of photosynthetic organisms, the endogenous sources of organic matter are mainly of bacterial and animal origin (decomposing products and bat droppings). Exogenous organic matter can be brought inside the cave through water runoff.

On the other hand, bat flies (Nycteribiidae and Streblidae), like all members of the superfamily Hippoboscoidea, reproduce via viviparous puparity. Thus, immature stages, from eggs to L3 larvae, develop in the uterine cavity until larviposition and progression from L3 larvae to pupae. Pupae that are not motile are found on rock walls or vaults close to their bat hosts until adult life.^16^

#### Blood source availability

Like for the immature stages, adults of hematophagous dipterans have developed physiological and behavioral adaptations to the specific environmental conditions inside caves (that is, temperature and relative humidity) and to the vertebrate hosts used as blood sources. Among mammals, bats and rodents are the main vertebrates found in Afrotropical caves.^17^ Bats form very large colonies and live in high promiscuity, thus providing an unlimited blood source. More than 300 species from 70 genera have been reported in the Afrotropical region and at least 95 species from 34 genera, including Yangochiroptera and Yinpterochiroptera (for example, *Miniopterus* sp.*, Rousettus* sp.*, Hipposideros* sp.*, Coleura* sp.*, Rhinolophus* sp.*, Pipistrellus* sp.*, Nycteris* sp.), roost in caves.^18^ All these bat species can potentially serve as blood sources for cave-dwelling dipterans, although only some species have been clearly identified as such.^19, 20^ The African porcupine (*Atherurus africanus* Gray) and rodents of the genus *Praomys* are the main taxa bitten by hematophagous cave-dwelling dipterans in African caves.^17^

#### Ecological classification and spatial occupancy of Diptera inside caves

The current classification of the cave fauna is based on the ecological criteria proposed by earlier speleologists in which species are grouped in three categories in relation with the degree of penetration in subterranean areas: trogloxenic, troglophilic and troglobitic. Trogloxenic dipterans are accidental, occasional or regular visitors that penetrate in caves for physiological reasons, associated with seasonal climatic variations (refuge during the dry season) or with resource gathering, but that do not complete their entire life cycle inside the cave.^21^ Troglophilic species visit and usually breed inside caves, but can be found also in epigeous areas at all stages.^21^ Troglobitic dipterans are exclusively confined in subterranean areas for their entire life cycle and their presence in epigeous area is exceptional and most often fatal.^21^

In caves, dipterans colonize all spaces, including those inaccessible to humans. Adults of flying Nematocera (for example, mosquitoes, sand flies or biting midges) rest on walls, vaults or crevices in rock walls and more rarely on soil or at the bottom of rocks. Sand flies prefer places with low air flow and are frequently observed inside secondary chambers, away from high air streams.^22^ Blood-engorged female dipterans are regularly observed close to bats or near rodent holes, whereas males and unfed teneral females occupy the area close to the breeding site or cave walls.^22^ Conversely, adult bat flies live mostly on the body of bats.^23^

### What is known about the diversity of blood-feeding Diptera in Afrotropical caves?

Cave-dwelling Diptera remain poorly known worldwide and the very few studies dedicated to cave-dwelling hematophagous species only reported a limited number of taxa among mosquitoes, sand flies,^24^ biting midges^25^ and bat flies.^26^ Most of the species of medical/veterinary interest were found in Afrotropical caves.^6^

#### Mosquitoes (Culicidae)

Among the almost thousand mosquito species of the Afrotropical region, only twelve have been described as cavernicolous. Because of their role in bat malaria transmission, species of the *Anopheles* (*An.*) genus have been more studied in some African countries, including the Democratic Republic of Congo (DRC), the Republic of Congo and occasionally Cameroon, Central African Republic (CAR), Gabon, Guinea and Madagascar (Table 1). Besides the *Anopheles* genus, only *Uranotaenia* (*Ur*.) *cavernicola* Mattingly in DRC, *Ur. fusca* Theobald and *Mansonia uniformis* Theobald in Madagascar were detected in caves (Table 1).

#### Sand flies (Phlebotominae)

The current knowledge on cave-dwelling sand flies in Africa is mainly based on studies carried out in Central, West and East Africa that reported the presence of several genera: *Spelaeophlebotomus* (*Sl.*), *Phlebotomus* (*Ph.*), *Sergentomyia* (*Se.*) and *Spelaeomyia* (*Sa.*). *Sl. gigas* Parrot and Schwetz (Table 1) was one of the first species discovered in caves. Although for a long time this species was thought to be endemic in DRC, it was subsequently found throughout Central and West Africa.^27^ Further investigations reported other cave-dwelling sand fly species of the genera *Phlebotomus* (*Ph.*), *Sergentomyia* (*Se.*) and *Spelaeomyia* (*Sa.*) in the continent (Table 1).

#### Biting midges (Ceratopogonidae)

Among biting midges, hematophagous species mainly belong to the genus *Culicoides* and, to a lesser extent, to the genera *Forcipomyia* and *Leptoconops*. These species have been largely documented in epigeous areas in South, East and West Africa, while they remain poorly studied in Central Africa.^28^ Although few studies have been undertaken in some caves in Central Africa, biting midges still remain under-documented in cave environments across the continent. Nevertheless, more than twenty species have been detected in caves, including fifteen new descriptions, but only three are hematophagous (Table 1), and their biology still remains unknown.

#### Bat flies (Nycteribiidae and Streblidae)

About 40 species and sub-species of Nycteribiidae and 32 species and sub-species of Streblidae are known in Africa.^29, 30^ Most of them parasitize epigeous bats in the Congo Basin (Central Africa) and southern Africa. Bat flies have been recorded also in Nigeria (West Africa), Cameroon (Central Africa), Kenya, Sudan, Tanzania and Uganda (East Africa).^29^ Specific studies on bat flies that parasitize cave-dwelling bats remain rare across the African continent and only six species have been described so far (Table 1). Two Nycteribiidae species (*Penicilidia fulvida* Bigot and *Nycteribia schmidlii scotti* Falcoz) parasitize mainly cave-dwelling bats of the genera *Miniopterus* (Republic of Congo, Gabon) and *Rhinolophus* (Republic of Congo).^20, 31^ *Eucampsipoda africana* Theodor infests the cave-dwelling *Rousettus aegyptiacus* Geoffroy in South Africa and Gabon.^20, 32^ Three Streblidae species (*Raymondia simplex* Jobling*, R. seminuda* Jobling and *Raymondioides leleupi* Jobling) have been found on *Miniopterus* and *Rhinolophus* bats that live in caves of the Republic of Congo.^31^ In Gabon, *Raymondia huberi* Frauenfeld and *Brachytarsina allaudi* Falcoz infest the bat species *Hipposideros caffer* Sundevall and *Hipposideros gigas* Wagner, respectively.^20^

### What is known about the vector role of blood-feeding Diptera in Afrotropical caves?

In tropical caves, several thousand bats often aggregate in very large roosts, thus favouring the circulation of pathogens, including many viruses, such as Lyssaviruses, Filoviruses, Coronaviruses, Paramyxoviruses or Flaviviruses.^33^ The presence of bat-biting dipterans in cave ecosystems increases the risk of arthropod-borne pathogen transmission because these insects are known vectors of blood parasites (for example, mosquitoes, sand flies and biting midges), viruses (for example, mosquitoes, sand flies and biting midges), bacteria (for example, mosquitoes and sand flies) and filarial parasites (for example, mosquitoes and biting midges).

#### Vector-borne parasites

*Haemosporidia*. Several arthropod-borne haemosporidian parasites have been identified in African cave-dwelling hosts, including rodents and bats. Among Plasmodidae, *Plamsodium atheruri* Van Den Berghe, Lambrecht and Zaghi, which infects African porcupines, has been found in the salivary glands of *An. smithii* complex members in caves of Cameroon^34^ and Ghana.^35^ *An. vanthieli* Laarman and *An. caroni* Adam were also identified as vectors of this parasite in Republic of Congo.^18^ However *An. hamoni* Adam is a suspected vector of *P. atheruri* and other blood parasites of the genera *Hepatocystis* and *Nycteria.*^18^ Members of the *An. smithii* complex are vectors of *Plasmodium voltaicum* Van der Kaay, a parasite that infects the bats *Lyssonycteris smithii* Thomas in Ghana and *Lyssonycteris angolensis* Bocage in the Republic of Congo.^36, 37^ Although their role has never been formally demonstrated, cave-dwelling *Anopheles* are also suspected to transmit *Plasmodium roussetti* Van Riel that infects the fruit bat *Rousettus aegyptiacus*.^17^

In caves, sand flies are mostly known for their role in the epidemiology of saurian Haemosporidia.^38^ In Africa, about twenty *Plasmodium* species have been detected in reptiles.^39^ Therefore the presence of various reptiles in caves could suggest a possible role of sand flies in saurian Haemosporidia circulation in these environments.

In the Afrotropical region, biting midges are considered the main vectors of bat *Hepatocystis* parasites in epigeous environments.^40^ However, their role in the transmission of *Hepatocystis* and other parasites, such as *Nycteria,* inside caves is only suspected, but not formally demonstrated.^17^

After *Anopheles* mosquitoes, bat flies are the most documented vectors of bat malaria parasites in caves. *Polychromophilus* parasites mainly infect bats of several genera, including *Miniopterus, Rhinolophus, Myotis, Pipistrellus, Hipposideros, Antrozous* and *Glosophaga*.^17^ Most of these bats are found in Afrotropical caves, especially the species *Miniopterus minor minor* Peters*, Rhinolophus sylvestris* group Aellen and *Hipposideros caffer* that are infected by *Polychromophilus* almost permanently.^41^ Recent investigations in Gabon caves revealed that the bat fly species *P. fulvida* and *Nycteribia schmidlii scotti* are heavily infected by *Polychromophilus melanipherus.* Moreover, the finding that both bat fly species parasitize *Miniopterus inflatus* Thomas, a natural host of this parasite,^20, 42^ suggests that these bat fly species might transmit this parasite. Infections by *P. melanipherus* have also been observed in *Eucampsipoda africana,* a Nycteribiidae species that infests cave-dwelling bats.^20^ To date, flies from the Streblidae family have never been incriminated in the transmission of haemosporidian parasites. Nevertheless, their vector role should be reinvestigated because *P. melanipherus* was molecularly detected in the streblid species *Brachytarsina allaudi* and *Raymondia huberi*.^20^ Moreover, infecting stages of a bat malaria parasite (*Vetufebrus ovatus* Poinar) were previously observed in a streblid specimen.^43^

*Filarial, leishmanial and trypanosomatid parasites.* Bats are important vertebrate hosts of filarial nematodes. Thirty-four genera of nematodes have been described^44^ and *Litomosa* and *Litomosoides* (Onchocercidae) are the best known.^45^ In Africa, bats, including cave-dwelling taxa,^46^ are infected mainly by parasites of the *Litomosa* genus, but few cases of infections by *Litomosoides* have also been reported.^45^ Infections by *Litomosa* spp. have been detected in several bat families.^47^ The vectors remain unknown, although mites (Macronyssidae)^48^ are suspected, as well as other dipterans, including Culicidae, Phlebotominae and Ceratopogonidae.

Sand flies are well-recognized vectors of *Leishmania* parasites in the tropics. Recent studies in caves of the Neotropical region (also called South American region) revealed that bats are infected by *Leishmania* sp.^49^ In Africa, cave-dwelling bats are important natural hosts of *Leishmania* parasites;^50^ however, the role of sand flies as vectors remains to be confirmed, despite their frequent co-occurrence with bats in caves.

Trypanosomatid parasites circulate among cave-dwelling reservoirs, including bats and rodents, in the Afrotropical region.^51^ The transmission modalities remain under-investigated. In the Neotropical region, sand flies are known vectors of trypanosomatid parasites.^52^ Conversely, in the Afrotropical region only the genus *Spelaeomyia emilii* Parrot and Wanson is suspected to be involved in the transmission of *Trypanosoma vespertilionis* Battaglia that infects cave-dwelling bats in the Republic of Congo.^51^ Besides sand flies, mosquitoes, particularly within the genus *Culex,* are suspected to be vectors of avian,^53^ amphibian^54^ and reptilian^55^ trypanosomatid parasites.

#### Viruses

Currently, it is not clear how hematophagous arthropods are involved in the circulation of viruses in cave ecosystems due to the limited number of studies on this question. Cave-dwelling vertebrates, including bats, are recognized hosts of a large diversity of zoonotic viruses, such as Paramyxoviruses, Lyssaviruses, Coronaviruses, Filoviruses, Hantaviruses and particularly arboviruses (for instance, the Flaviviridae, Bunyaviridae and Reoviridae families).^33^ Some of them cause diseases in humans or in domestic animals.^56, 57^ Most of the arboviruses or potential arboviruses found in African bats have been reported or isolated from the bat genera *Nycteris, Miniopterus* or *Hipposideros*.^33^ Considering the major role of dipterans, such as mosquitoes, sand flies, bat flies or even biting midges, in the transmission of bat arboviruses,^58^ it would be of great interest to thoroughly investigate the epidemiology of arboviruses in cave ecosystems, by carrying out arbovirus screening in cave-dwelling bats and hematophagous dipterans.

#### Bacteria

Little is known about the role of hematophagous insects in the epidemiology of bacteria that infect wild mammals living in African caves. Among the known arthropod-borne bacteria, *Bartonella* spp. is one of the most widely transmitted among vertebrates, including bats.^59^ In cave environments, bats are the main hosts of bacteria and bacteria are mainly transmitted by bat ectoparasites, such as ticks (Arachnida) and bat flies.^60^ In Africa, recent studies described *Bartonella* spp. infections in the cave-dwelling bats *Eidolon helvum* Kerr and *E. dupreanum* Schlegel and Pollen, and showed the potential role of the Nycteribiidae *Cyclopodia greefi greefi* Karsch and *Cyclopodia dubia* Westwood in their transmission.^61, 62^ Although the *Bartonella* species were not identified, previous phylogenetic investigations highlighted the strong similarity between the *Bartonella* spp. found in bat flies and in bats, suggesting that bat flies might be the involved vectors.^63^

Like bat flies, sand flies also are *Bartonella* spp. vectors.^64^ Although *Bartonella* spp. circulate in Afrotropical cave ecosystems,^59^ sand fly role in their transmission has not been investigated yet.

*Borrelia* bacteria infect different bat species worldwide,^60^ but little is known about African bats. Ticks are the primary vectors of *Borrelia* bacteria^65^ and several mosquito genera, such as *Anopheles*, *Culex* and *Aedes,* are suspected to ensure their transmission.^66^

### Challenges and future research directions

The circulation of a wide range of pathogens hosted by vertebrate reservoirs in cave environments constitutes a potential major hazard for the health of humans and domestic animals because of the cave progressive anthropization. Some previous studies have shown that some species of sand flies (for example, *Sl. gigas*) and mosquitoes (for example, *An. hamoni* and *An. caroni*) are opportunistic and can feed on bats, rodents, and occasionally humans.^10, 67^ Some caves that host large communities of hematophagous dipterans are located in villages, towns, and even in farming concessions.^19^ This can increase the contact between cavernicolous hematophagous dipterans and humans, thus favouring the accidental transmission of pathogens to humans or livestock. Such contacts and transfers could occur when people or domestic animals enter inside caves, but also via trogloxenic dipteran species that could bring pathogens from inside the cave to the cave surroundings, which may include areas inhabited by human populations. Depending on the pathogen and on the host susceptibility, a new pathogen could emerge in humans and cause local outbreaks, if it can find a suitable vector to ensure its epidemic transmission among humans. Due to their ability to transmit some pathogens, cave-dwelling hematophagous dipterans are of important concern for researchers and health authorities. Among the Diptera-related factors that contribute to the zoonotic hazard of cave environments, the most important are: (i) the ability to support natural enzootic and intra-specific pathogen cycles that involve a natural primary reservoir. This could increase the risk of opportunistic or accidental inter-specific transmission to humans or domestic animals; and (ii) the ability of cave-dwelling hematophagous dipterans, particularly trogloxenic insects, to bite humans or domestic animals, even accidentally. However, contacts between cave fauna and humans are not yet widespread and general, are still restricted to the surrounding populations and only occasionally to far-away victims.

To date, little is known about Diptera ecology and their vector role in Afrotropical caves. Indeed, basic data were collected several decades ago by few passionate researchers who made substantial efforts to overcome the inaccessibility and hostile environmental conditions of African caves. This pioneering research was not further pursued, although many questions remain unanswered about the transmission cycles involving arthropod vectors. Recent technical advances (that is, molecular biology and high-throughput sequencing tools for vector taxonomy and pathogen screening) could certainly help filling in these gaps. The renewed interest on cave-dwelling Diptera and associated pathogens, as attested by recent studies in Gabon, provided new insights into the diversity of cave-dwelling insects of medical importance.^20^ The discovery of several pathogens in cave-dwelling reservoirs using advanced molecular tools^68^ should help assessing the risk of transfer to humans or domestic animals that live in the vicinity of caves. Indeed pathogen surveillance of wild and domestic animals in cave-associated areas would improve our knowledge on the transmission risk as well as on arthropod-borne pathogens and their associated vectors. Studies on parasite ecology, such as their life cycle and the gonotrophic cycle of vectors, could bring crucial information on how these bioecological features influence pathogen epidemiology in cave-associated areas. Extending research to caves of new areas and to other arthropod groups of medical and veterinary importance, such as bugs, fleas and ticks, will provide integrated data on the diversity, spatial distribution and role in pathogen epidemiology of cave-dwelling hematophagous arthropods.

## Figures and Tables

**Table 1:** Known cave-dwelling hematophagous Diptera in the Afrotropical region

**Species**	**Site**	**Country**	**Vector role**	**References**
*Mosquitoes*				
* An. vanhoofi*	Mbanza-Ngungu	DRC	Suspected vector of *Plasmodium* spp.	^18, 69^
* An. rodhaini*	Likasi	DRC	Suspected vector of *Plasmodium* spp.	^18, 70^
* An. faini*	Yolaha firi	DRC	Suspected vector of *P. roussetti*	^18, 71^
* An. smithii rageaui*	Oliga	Cameroon	Vector of *P. atheruri* and *P. voltaicum*	^18, 36, 37, 72^
* An. cavernicolus*	Dalaba region	Guinea	Unknown	^73^
* An. vanthieli*	Irangi	DRC	Vector of *P. atheruri*	^18, 74^
* An. caroni*	Matouridi	Congo-Brazzaville	Vector of *P. atheruri*	^18, 75^
* An. hamoni*	Meya-Nzouari	Congo-Brazzaville	Suspected vector of *Plasmodium* spp. *Hepatocystis* spp.*, Nycteria* spp.	^18, 76^
* An. pauliani*	Aven Anjohy	Madagascar	Unknown	^77^
* Ur. cavernicola*	Yoloha firi	Congo-Brazzaville	Unknown	^78^
* Ur. fusca*	Bemaraha	Madagascar	Unknown	^77^
* Ma. uniformis*	Bemaraha	Madagascar	Unknown	^77^

*Sand flies*				
* Sl. gigas*	Matadi	DRC	Unknown	^27^
* Sa. emilii*	Loudima	Congo-Brazzaville	Suspected vector of *Trypanosoma vespertilionis*	^27, 51^
* Sa. darlingi*	Jabel Tozi	Sudan	Unknown	^27^
	Sikasso	Mali	Unknown	^27^
* Sa. moucheti*	Koumba	Cameroon	Unknown	^27^
	Bébé	CAR	Unknown	^27^
* Se. mirabilis*	Mbanza-Ngungu	DRC	Vector of *Leishmania* spp., *Trypanosoma* spp.	^10, 27^
* Se. balmicola*	Akok-Bekue	Cameroon	Unknown	^27^
* Se. africana*	Muruku	Kenya	Unknown	^79^
* Se. bedfordi*	Muruku	Kenya	Unknown	^79^
* Ph. somaliensis*	Shama-Aleh	Somalia	Unknown	^27^

*Biting midges*				
* C. brossetti*	Faucon	Gabon	Unknown	^80^
* C. grenieri*	Meya-Nzouari	Congo-Brazzaville	Unknown	^80^
* C. rageaui*	Meya-Nzouari	Congo-Brazzaville	Unknown	^80^

*Bat flies*				
* P. fulvida*	Undetermined	Congo-Brazzaville	Suspected vector of *P. melanipherus*	^20, 51^
	Kessipoughou, Djibilong, Faucon	Gabon		^20^
* N. schmidlii scotti*	Undetermined	Congo-Brazzaville	Suspected vector of *P. melanipherus*	^20, 51^
	Kessipoughou, Djibilong, Faucon	Gabon		^20^
* E. africana*	Mahoume	South Africa	Unknown	^32^
	Kessipoughou, Zadie	Gabon	Unknown	^20^
* R. simplex*	Undetermined	Congo-Brazzaville	Unknown	^51^
* R. seminuda*	Undetermined	Congo-Brazzaville	Unknown	^51^
* R. huberi*	Kessipoughou, Djibilong	Gabon	Unknown	^20^
* B. allaudi*	Kessipoughou	Gabon	Unknown	^20^
* R. leleupi*	Undetermined	Congo-Brazzaville	Unknown	^51^

In these studies, flying dipterans (mosquitoes, sand flies and biting midges) were collected manually on cave walls or using light traps. Bat flies were collected manually on captured bats.

## References

[bib1] Abadia OM, Gonzalez Morales MR. Paleolithic art: a cultural history. J Archaeol Res 2013; 21: 269–306.

[bib2] Nieves-Rivera AM, Santos-Flores CJ, Dugan FM, Miller TE. Guanophilic fungi in three caves of southwestern Puerto Rico. Int J Speleol 2009; 38: 71–82.

[bib3] Plowright RK, Eby P, Hudson PJ et al. Ecological dynamics of emerging bat virus spillover. Proc R Soc Lond B Biol Sci 2015; 282: 20142124. 10.1098/rspb.2014.2124 PMC4262174 25392474

[bib4] Reeves WK, Loftis AD, Gore JA, Dasch GA. Molecular evidence for novel *Bartonella* species in *Trichobius major* (Diptera: Streblidae) and *Cimex adjunctus* (Hemiptera: Cimicidae) from two southeastern bat caves, USA. J Vector Ecol 2005; 30: 339–341. 16599175

[bib5] Singh B, Daneshvar C. Human infections and detection of *Plasmodium knowlesi*. Clin Microbiol Rev 2013; 26: 165–184. 23554413 10.1128/CMR.00079-12 PMC3623376

[bib6] Matile L. Les Diptères cavernicoles. Ann speleol 1970; 25: 179–222.

[bib7] Fain A. Notes sur les Punaises parasites de Chiroptères de la République du Zaïre avec description de deux espèces et d'une sous-espèce nouvelles. Rev Zool Bot Afr 1972; 85: 187–202.

[bib8] Sondag F, Van Ruymbeke M, Soubiès F et al. Monitoring present day conditions in tropical caves using an environmental data acquisition system (EDAS). J Hydrol 2003; 273: 103–118.

[bib9] Gamble DW, Dogwiler JT, Mylroie J. Field assessment of the microclimatology of tropical flank margin caves. Clim Res 2000; 16: 37–50.

[bib10] Vattier-Bernard G. Contribution à l'étude systématique et biologique des phlébotomes cavernicoles en Afrique intertropicale (2ème partie). Cah ORSTOM Ser Ent Med Parasitol 1970b; 8: 231–288.

[bib11] Lignier V. Éléments de Karstologie et Géologie pour Spéléo - Stage perfectionnement du CDS 69 2008; Available at http://cds69.free.fr/wp-content/uploads/Rapport-karstologie-Vincent-Lignier.pdf. French.

[bib12] Adam JP. Rapport sur une mission au Gabon pour l'étude préliminaire de la faune de quelques grottes de la région de Makokou (18 au 31 janvier 1966) 1966; Available at http://www.documentation.ird.fr/hor/fdi:10541. French.

[bib13] Van Someren ECC, Mutinga MJ. Interesrting mosquito records from Kenya. Mosq Syst Newslett 1971; 3: 211.

[bib14] Vattier-Bernard G, Adam JP. Les Ceratopogonidae des grottes de la République du Congo. Ann Speleol 1966; 21: 711–773.

[bib15] Vattier-Bernard G. Etudes morphologique et biologique des phlébotomes cavernicoles du Congo-Brazzaville. Ann Speleol 1971; 26: 149–171.

[bib16] Dittmar K, Dick CW, Patterson BD, Whiting MF, Gruwell ME. Pupal deposition and ecology of bat flies (Diptera: Streblidae): *Trichobius* sp. (*caecus* group) in a Mexican cave habitat. J Parasitol 2009; 95: 308–314. 18684039 10.1645/GE-1664.1

[bib17] Adam JP. Les hémosporidies parasites d'animaux cavernicoles, Fonds IRD fdi:28690, 1974. Available at http://www.documentation.ird.fr/hor/fdi:28690. French.

[bib18] African Chiroptera Report (ACR). African Bats, Pretoria 2014. Available at www.africanbats.org (accessed on 4 August 2014).

[bib19] Adam JP. [Transmission of haemosporidia by *Anopheles* mosquitoes in the caves of Congo (Brazzaville)]. Bull World Health Organ 1965; 32: 598–602. 14315730 PMC2555257

[bib20] Obame-Nkoghe J, Rahola N, Bourgarel M et al. Bat flies (Diptera: Nycteribiidae and Streblidae) infesting cave-dwelling bats in Gabon: diversity, dynamics and potential role in *Polychromophilus melanipherus* transmission. Parasit Vectors 2016; 9: 333. 27286888 10.1186/s13071-016-1625-z PMC4902993

[bib21] Vandel A. Biospéléologie: La Biologie des Animaux Cavernicoles. Gauthier-Valliars (ed), Paris, 1964, 619.

[bib22] Vattier-Bernard G. Notes sur la biologie de deux espèces de phlébotomes cavernicoles africains. Bull Soc Ecol 1971; 2: 293–301.

[bib23] Dick CW, Patterson BDBatflies - Obligate ectoparasites of bats. In: Morand S, Krasnov BR, Poulin R (eds). Micrommals and Macroparasites From Evolutionary Ecology to Management. Springer-Verlag: Tokyo, Japan, 2006, 179–196.

[bib24] Alves VR, Freitas RA, Santos FL, Barrett TV. Diversity of sandflies (Psychodidae: Phlebotominae) captured in sandstone caves from Central Amazonia, Brazil. Mem Inst Oswaldo Cruz 2011; 106: 353–359. 21655825 10.1590/s0074-02762011000300016

[bib25] Wirth WW. The biting midges of the Batu caves, Malaysia (Diptera: *Ceratopogoniade*. Pacific Insects 1980; 21: 304–307.

[bib26] Maa TC. Records and descriptions of Nycteribiidae and Streblidae (Diptera). Pacific Insects 1962; 4: 417–436.

[bib27] Vattier-Bernard G, Adam JP. Connaissances actuelles sur la répartition géographique des phlébotomes cavernicoles africains; Considérations sur l'habitat et la biologie. Ann Speleol 1969; 24: 143–161.

[bib28] Itoua A, Cornet M, Vattier-Bernard G, Trouillet J. Les Ceratopogonidae (Diptera, Ceratopogonidae) d'Afrique Centrale. Cah ORSTOM Ser Entomol Med Parasitol 1987; 25: 127–134.

[bib29] Theodor O. An Illustrated Catalogue of the Rothschild Collection of Nycteribiidae (Diptera) in the British Museum (National History) with Keys and Short Descriptions for the Identification of Subfamilies, Genera, Species and Subspecies. Trustees of the British Museum (Natural History): London, 1967.

[bib30] Theodor O. A revision of the Streblidae (Diptera) of the Ethiopian Region. Trans R Entomol Soc Lond 1968; 120: 313–373.

[bib31] Adam JP, Landau I. Developmental stages of *Polychromophilus* sp., a parasite of insectivorous bats from the Congo-Brazzaville, in the nycteribiid fly *Penicillidia fulvida* Bigot 1889. Trans R Soc Trop Med Hyg 1973b; 67: 5–6. 10.1016/0035-9203(73)90260-5 4777434

[bib32] Jansen van Vuren P, Wiley M, Palacios G et al. Isolation of a Novel Fusogenic *Orthoreovirus* from *Eucampsipoda africana* Bat Flies in South Africa. Viruses 2016; 8: 65–89. 27011199 10.3390/v8030065 PMC4810255

[bib33] Maganga GD, Bourgarel M, Vallo P et al. Bat distribution size or shape as determinant of viral richness in african bats. PLoS One 2014; 9: e100172. 24959855 10.1371/journal.pone.0100172 PMC4069033

[bib34] Mouchet J, Gariou J, Rivola E. [Observations on the biology of *Anopheles smithii* var. *rageaui* Mattingly and Adam 1954, vector of a mammalian *Plasmodium* in the outskirts of Yaoundé (southern Cameroon)]. Bull Soc Pathol Exot 1957; 50: 157–164. 13437128

[bib35] Brady J. The occurrence of Anopheles smithii var. rageaui Mattingly and Adam in Ghana, with a note on its possible implication as a vector of non-human malaria. Ann Trop Med Parasitol 1965; 59: 99–105. 14297362 10.1080/00034983.1965.11686288

[bib36] Adam JP Transission d'hémosporides chez les animaux cavernicoles International Congress of Parasitology. Vienne: Facta Publication 1974, 85–86.

[bib37] Adam JP, Landau I. Plasmodium voltaicum au Congo-Brazzaville. J Parasitol 1970; 56: 391–393.

[bib38] Ayala SC, Lee D. Saurian malaria. Development of sporozoites in 2 species of phlebotomine sandflies. Science 1970; 167: 891–898. 5410856 10.1126/science.167.3919.891

[bib39] Mutinga MJ, Dipeolu OO. Saurian malaria in Kenya. Epidemiologic features of malarial infections in lizard populations of the Pokot district. Int J Parasitol 1990; 20: 149–153. 2332274 10.1016/0020-7519(90)90094-4

[bib40] Landau I. Diversité des mécanismes assurant la pérennité de l'infection chez les sporozoaires coccidiomorphes. Mem Mus Natl Hist Nat Paris A Zool 1973; vol. 77: 62 pp.

[bib41] Adam JP, Landau I. *Polychromophilus* sp. Hemoproteidea parasite of Microchiroptera in Congo (Brazzaville). Cah Orstom Ser Ent Med Parasitol 1973; 11: 147–152.

[bib42] Duval L, Mejean C, Maganga GD et al. The chiropteran haemosporidian *Polychromophilus melanipherus*: A worldwide species complex restricted to the family Miniopteridae. Infect Genet Evol 2012; 12: 1558–1566. 22721902 10.1016/j.meegid.2012.06.006

[bib43] Poinar GO Jr. *Vetufebrus ovatus* n. gen., n. sp. (Haemospororida: Plasmodiidae) vectored by a streblid bat fly (Diptera: Streblidae) in Dominican amber. Parasit Vectors 2011; 4: 229–233. 22152687 10.1186/1756-3305-4-229 PMC3253689

[bib44] Morand S, Bouamer S, Hugot JP. Nematodes. In: Morand S, Krasnov BR, Poulin R (eds). Micromammals Macroparasites: From Evolutionary to Ecology Managment. Tokyo: Springer-Verlag, 2006; 63–79.

[bib45] Ramasindrazana B, Dellagi K, Lagadec E et al. Diversity, Host Specialization, and Geographic Structure of Filarial Nematodes Infecting Malagasy Bats. PLoS One 2016; 11: 1–18. 10.1371/journal.pone.0145709 PMC4709050 26751792

[bib46] Martin C, Bain O, Jouvenet N et al. First report of *Litmosa* spp. (Nematoda: Filarioidea) from Malagasy bats; review of the genus and relationships between species. Parasite 2006; 13: 3–10. 16605061 10.1051/parasite/2006131003

[bib47] Raharimanga V, Ariey F, Cardiff SG et al. Hemoparasites of bats in Madagascar. Arch Inst Past Mad 2003; 69: 70–76. 15678820

[bib48] Guerrero R, Bain O, Attout T, Martin C. The infective larva of *Litomosoides yutajensis* Guerrero et al., 2003 (Nematoda: Onchocercidae), a Wolbachia-free filaria from bat. Parasite 2006; 13: 127–130. 16800120 10.1051/parasite/2006132127

[bib49] Ogawa GM, Pereira Junior AM, Resadore F et al. Sandfly fauna (Diptera: Psychodidae) from caves in the state of Rondonia, Brazil. Rev Bras Parasitol Vet 2016; 25: 61–68. 27007243 10.1590/S1984-29612016017

[bib50] Kassahun A, Sadlova J, Benda P et al. Natural infection of bats with *Leishmania* in Ethiopia. Acta Trop 2015; 150: 166–170. 26232657 10.1016/j.actatropica.2015.07.024

[bib51] Adam JP. Hématozoaires des chiroptères en Afrique Centrale. Cah Orstom Ser Ent Med Parasitol 1973; 6075: 2–7.

[bib52] Zeledon R, Rosabal R. *Trypanosoma Leonidasdeanei* sp. nov. in insectivorous bats of Costa Rica. Ann Trop Med Parasitol 1969; 63: 221–228. 5392759 10.1080/00034983.1969.11686623

[bib53] Votypka J, Szabova J, Radrova J, Zidkova L, Svobodova M. *Trypanosoma culicavium* sp. nov., an avian trypanosome transmitted by *Culex* mosquitoes. Int J Syst Evolut Microbiol 2012; 62: 745–754. 10.1099/ijs.0.032110-0 21515704

[bib54] Bartlett-Healy K, Crans W, Gaugler R. Vertebrate hosts and phylogenetic relationships of amphibian trypanosomes from a potential invertebrate vector, *Culex territans* Walker (Diptera: Culicidae). J Parasitol 2009; 95: 381–387. 18850768 10.1645/GE-1793.1

[bib55] Telford SR. Haemoparasites of reptiles. In: Hoff GL, Frye FL, Jacobson ER (eds). Diseases of Amphibians and Reptiles. New York, NY: Plenum Press, 1984: 385–517.

[bib56] Chua KB, Crameri G, Hyatt A et al. A previously unknown reovirus of bat origin is associated with an acute respiratory disease in humans. Proc Natl Acad Sci USA 2007; 104: 11424–11429. 17592121 10.1073/pnas.0701372104 PMC1899191

[bib57] Faburay B, Wilson WC, Gaudreault NN et al. A Recombinant Rift Valley Fever Virus Glycoprotein Subunit Vaccine Confers Full Protection against Rift Valley Fever Challenge in Sheep. Sci Rep 2016; 6: e277719. 10.1038/srep27719 PMC4906348 27296136

[bib58] Melaun C, Werblow A, Busch MW, Liston A, Klimpel S. Bats as potential reservoir hosts for vector-borne diseases. In: Klimpel S, Mehlhorn H (eds). Bats (Chiroptera) as Vectors of Diseases and Parasites. Berlin: Springer, 2014: 25–61.

[bib59] Kosoy M, Bai Y, Lynch T et al. *Bartonella* spp. in bats, Kenya. Emerg Infect Dis 2010; 16: 1875–1881. 21122216 10.3201/eid1612.100601 PMC3294596

[bib60] Mühldorfer K. Bats and bacterial pathogens: a review. Zoonoses Public Health 2013; 60: 93–103. 22862791 10.1111/j.1863-2378.2012.01536.x

[bib61] Billeter SA, Hayman DTS, Peel AJ et al. *Bartonella* species in bat flies (Diptera: Nycteribiidae) from western Africa. Parasitology 2012; 139: 324–329. 22309510 10.1017/S0031182011002113

[bib62] Brook CE, Bai Y, Dobson AP et al. *Bartonella* spp. in Fruit Bats and Blood-Feeding Ectoparasites in Madagascar. PLoS Neglect Trop Dis 2015; 9: e0003532. 10.1371/journal.pntd.0003532 PMC4337899 25706653

[bib63] Morse SF, Olival KJ, Kosoy M et al. Global distribution and genetic diversity of *Bartonella* in bat flies (Hippoboscoidea, Streblidae, Nycteribiidae). Infect Genet Evol 2012; 12: 1717–1723. 22771358 10.1016/j.meegid.2012.06.009

[bib64] Billeter SA, Levy MG, Chomel BB, Breitschwerdt EB. Vector transmission of *Bartonella* species with emphasis on the potential for tick transmission. Med Vet Entomol 2008; 22: 1–15. 18380649 10.1111/j.1365-2915.2008.00713.x

[bib65] Burgdorfer W. Discovery of the Lyme disease spirochete and its relation to tick vectors. Yale J Biol Med 1984; 57: 515–520. 6516454 PMC2590008

[bib66] Magnarelli LA, Freier JE, Anderson JF. Experimental infection of mosquitos with *Borrelia burgdorferi*, the ethiologic agent of Lyme disease. J Infect Dis 1987; 156: 694–695. 3624914 10.1093/infdis/156.4.694

[bib67] Adam JP. Les Culicidae cavernicoles du Congo et de l'Afrique intertropicale. Ann Speleol 1965; 20: 409–423.

[bib68] Li L, Victoria JG, Wang C et al. Bat guano virome: predominance of dietary viruses from insects and plants plus novel mammalian viruses. J Virol 2010; 84: 6955–6965. 20463061 10.1128/JVI.00501-10 PMC2898246

[bib69] Wanson M, Lebied B. *Anopheles**Myzomyia**vanhoofi* sp. n. Rev Zool Bot Africaines 1945; 39: 199–229.

[bib70] Leleup N, Lips M. Un anophèle nouveau du Katanga. *Anopheles rodhaini*. Rev Zool Bot Africaines 1950; 43: 303–308.

[bib71] Leleup N. Un anophèle nouveau du Kibali-Itouri. *Anopheles faini* n. sp. Rev Zool Bot Africaines 1952; 46: 151–158.

[bib72] Mattingly PF, Adam JP. A new species of cave-dwelling anopheline from the french Cameroon. Ann Trop Med Parasitol 1954; 48: 55–57. 13149119 10.1080/00034983.1954.11685598

[bib73] Abonnenc E. Sur un anophèle cavernicole de la Guinée: *Anopheles cavernicolus* n. sp. (Diptera *Culicidae*. Bull Mem Ec Med Pharm Dakar 1954; 2: 288–290.

[bib74] Laarman JJ. A new species of *Anopheles* from a rainforest in eastern Belgian Congo. Trop Geogr Med 1959; 2: 147–156. 14413159

[bib75] Adam JP. *Anopheles caroni* n. sp. un anophèle (*Diptera Culicidae* cavernicole de la République du Congo. Bull Soc Path Exot 1961; 54: 714–717.

[bib76] Adam JP. Un anonphèle cavernicole nouveau de la République du Congo (Brazzaville): *Anopheles hamoni* n. sp. (Diptera-Culicidae). Bull Soc Path Exot 1962; 55: 153–163. 13859241

[bib77] Remillet M. Apperçu de la faune souterraine à Madagascar. In: Academiei Republicii Socialiste Romania (ed). Livre du Cinquantenaire de l'Institut de Spéléologie "Emile Racovitza". Colloque National de Spéléologie. Bucharest, 1973: 135–160.

[bib78] Mattingly PF. Notes on Ethiopian Uranotaenia (Diptera-Culicidae) with description of a new species. Proc R Ent Soc Lond B 1954; 23: 166–171.

[bib79] Lawyer PG, Mebrahtu YB, Ngumbi PM et al. Phlebotomus *Guggisbergi* (Diptera, Psychodidae), a vector of *Leishmania tropica* in Kenya. Am J Trop Med Hyg 1991; 44: 290–298. 2035750 10.4269/ajtmh.1991.44.290

[bib80] Vattier-Bernard G, Adam JP. Capture de Ceratopogonidae (Diptera) Dans les Grottes de la République Gabonaise. Fonds IRD fdi:29022, 1965. Available at http://www.documentation.ird.fr/hor/fdi:29022. French.

